# Life beneath the surface of the central Texan Balcones Escarpment: genus *Anillinus* Casey, 1918 (Coleoptera, Carabidae, Bembidiini): new species, a key to the Texas species, and notes about their way of life and evolution

**DOI:** 10.3897/zookeys.417.7733

**Published:** 2014-06-19

**Authors:** Igor M. Sokolov, James R. Reddell, David H. Kavanaugh

**Affiliations:** 1 Department of Entomology, California Academy of Sciences, 55 Music Concourse Drive, Golden Gate Park, San Francisco, CA 94118, U.S.A.; 2 Texas Memorial Museum, University of Texas, Austin, TX 78705-5730, U.S.A.

**Keywords:** Coleoptera, Adephaga, Carabidae, Bembidiini, Anillina, *Anillinus*, new species, Texas, cave faunas, soil faunas, Balcones Fault Zone, Edwards-Trinity aquifer, Lampasas Cut Plain

## Abstract

The Texas fauna of the genus *Anillinus* Casey, 1918 includes three previously described species (*A. affabilis* (Brues), 1902, *A. depressus* (Jeannel), 1963 and *A. sinuatus* (Jeannel), 1963) and four new species here described: *A. acutipennis* Sokolov & Reddell, **sp. n.** (type locality: Fort Hood area, Bell County, Texas); *A. comalensis* Sokolov & Kavanaugh, **sp. n.** (type locality: 7 miles W of New Braunfels, Comal County, Texas); *A. forthoodensis* Sokolov & Reddell, **sp. n.** (type locality: Fort Hood area, Bell County, Texas); *A. wisemanensis* Sokolov & Kavanaugh, **sp. n.** (type locality: Wiseman Sink, Hays County, Texas). A key for identification of adults of these species is provided. The fauna includes both soil- and cave-inhabiting species restricted to the Balcones Fault Zone and Lampasas Cut Plain and adjacent areas underlain by the Edwards-Trinity Aquifer. Based on morphological and distributional data, we hypothesize that four lineages of endogean *Anillinus* species extended their geographical ranges from a source area in the Ouachita-Ozark Mountains to the Balconian region in central Texas. There the cavernous Edwards-Trinity aquifer system provided an excellent refugium as the regional climate in the late Tertiary and early Quaternary became increasingly drier, rendering life at the surface nearly impossible for small, litter-inhabiting arthropods. Isolated within the Edwards-Trinity aquifer system, these anilline lineages subsequently differentiated, accounting for the currently known diversity. The paucity of specimens and difficulty in collecting them suggest that additional undiscovered species remain to be found in the region.

## Introduction

Representatives of the subtribe Anillina are typically litter- or soil-dwelling carabids, but they are not common in caves. Of the approximately 50 anilline species previously described from North America, only four species of *Anillinus* (namely, *Anillinus longiceps* Jeannel (1963a), *Anillinus smokiensis* Sokolov (2011), *Anillinus tombarri* Sokolov (2012), and *Anillinus valentinei* (Jeannel 1963b) and both described species of the genus *Anillaspis* Jeannel can be considered true troglobitic species. In North America, anilline beetles are more common and diverse in the wet and forested areas of the southern Appalachians (42 species of the 56 species known to date from the continent) than in the dry regions to the West of the Mississippi River (where only 14 species have been recorded) (Bousquet 2012; Sokolov 2012; Sokolov and Carlton 2012).

During the last 10 years, the caves of central Texas have been intensely monitored because of conservation issues involving endangered troglobitic species threatened by urban development (see U.S. Fish and Wildlife Service, Department of Interior 2000, Paquin and Hedin 2004, Ledford et al. 2012). Central Texas harbors one of the richer and more distinctive cave faunas of North America and the world (Culver et al. 2003, 2006). More than a thousand cavernicole taxa have been reported to date, including at least 160 cave obligates (troglobites) (Reddell 1994). Troglobitic species are known among schizomids, pseudoscorpions, myriapods, collembolans, thysanurans, diplurans, and crustaceans, but the most impressive radiations of cave obligates are found in spiders, opilionids, and beetles. Troglobitic Coleoptera are represented by Staphylinidae: Pselaphinae (Chandler 1992, Chandler and Reddell 2001, Chandler et al. 2009), Curculionidae (Paquin and Anderson 2009), and Carabidae, particularly members of the genus *Rhadine* (*subterranea* group) (Barr 1974, Reddell and Cokendolpher 2001, 2004, Reddell and Dupérré 2009). During these expeditions, in addition to typical cave dwellers, a small number of anilline carabids have been collected. In approximately 40 years of surveying Texas caves, only 40 specimens of Anillina have been found. Their rarity in collections may reflect habitat preferences that are unlike other troglobites because most surveys in Texas were carried out by experienced biospeleologists, namely the second author [JRR] and his collaborators, who are familiar with the collection of tiny arthropods.

Before the cave survey, the Texas fauna of anillines had included only three species of these tiny eyeless carabids, which were represented in collections by a total of only five specimens. Such diversity is low in comparison with anilline faunas of North Carolina (14), Tennessee (12), or Alabama (9) (Bousquet 2012; Sokolov 2012; Sokolov and Carlton 2012) and possibly reflects certain difficulties in sampling the endogean microfauna in general and anillines in particular.

The first anilline species from Texas was described as *Anillus affabilis* Brues (1902: 366) from three specimens “collected at Austin, Texas”. According to Brues, all three specimens were taken from ant nests: two of these specimens “were sifted from a nest of *Eciton coecum* Latreille [=*Labidus coecus*], and one from a nest of *Solenopsis geminata* Fabricius”. The author of the description did not consider the species myrmecophilous, but pointed out that the beetles evidently shared their habitats with ants. After that first description, no new species were described from Texas for more than 60 years and only this species was cited for Texas in the first edition of the Catalogue of the Coleoptera of North America (Leng 1920). One more species, *Anillus debilis* LeConte (1853), originally described from California, was added to the Texas fauna in the fourth supplement to Leng’s catalogue (Blackwelder 1939). Obviously erroneous, this reference was copied from Jeannel’s first treatment of endogean bembidiines (Jeannel 1937). In the latter paper, Jeannel transferred all North American anillines, including Texan species, to the genus *Anillinus* Casey. However, later, in his main monograph on the world Anillina (Jeannel 1963a), he changed his view on the taxonomy of the North American fauna. In that monograph, he proposed a multilevel generic arrangement of the world anillines in “divisions” and “séries phylétiques.” Unfortunately, his inability to distinguish between two common genera from the eastern USA, *Anillinus* and *Serranillus* Barr (see Sokolov et al. 2004), led him to interpret the North American anilline genera incorrectly. As a main distinguishing character among the North American lineages, Jeannel (1963a, p. 48) used the number of parameres (one or two) in male genitalia. Accordingly, representatives of *Anillinus*, which Jeannel considered to have only one paramere (“à un seul style”), were incorrectly contrasted with representatives of anillines with the normal aedeagal configurations (i.e., two parameres). We surmise that Jeannel was using members of a *Serranillus* species, males of which lack the right paramere, leaving just the left one (Barr 1995; Sokolov et al. 2004; Sokolov and Carlton 2008, 2012) as his exemplars for genus *Anillinus* rather than true *Anillinus* specimens when he made these comparisons. As a result, Jeannel conceived of two North American lineages whose ranges roughly corresponded to the regions to the East and West of the Mississippi River. For the western fauna of non-troglobitic anillines, a new genus *Anillodes* Jeannel was established, and *Anillodes affabilis* from Texas subsequently changed its taxonomic position for the second time. In addition, Jeannel described two new anillines from Texas, *Anillodes sinuatus* Jeannel and *Micranillodes depressus* Jeannel, each based exclusively on a single female. The holotype of the latter species supposedly possessed a labium with the mentum and submentum fused and had shortened elytra, which prompted Jeannel to erect a new genus for this species. This is how California and Texas anillines, technically both “western” but otherwise quite distinct from one another, came to be included in one genus, *Anillodes*. A few years ago, while preparing his revised Catalogue of Geadephaga of America, north of Mexico, (Bousquet 2012), Yves Bousquet examined types of the Texas species of Anillina and judged them to be congeneric with the eastern *Anillinus* species and returned *Anillinus affabilis* to that genus. He also transferred *Anillinus sinuatus* and *Micranillodes depressus* (l.c.) to *Anillinus*. Currently, therefore, the Texas anillines are represented by three species in the genus *Anillinus*: *Anillinus affabilis*, *Anillinus depressus* and *Anillinus sinuatus*.

Our preliminary investigation of the Texas Anillina collected in caves showed that material at hand could not be identified by reference to the existing original descriptions and, therefore, more thorough taxonomic study was needed. This paper presents the results of our taxonomic study of all the Texas representatives of Anillina known to the authors to date. The question of relationships between *Anillinus* and *Anillodes* is beyond the scope of this paper.

## Material and methods

This study is based on examination of 39 specimens of Anillina from Texas, representing seven species, four of which are described as new. Most of the specimens originated from the collection of the Texas Memorial Museum, The University of Texas at Austin, Austin, Texas, USA (TMM). Types and a few additional specimens were borrowed from the U. S. National Museum of Natural History (NMNH), Smithsonian Institution, Washington, DC, and the Museum of Comparative Zoology (MCZ), Harvard University, Cambridge, MA. Specimens from this study, including types, have also been deposited in collections at the California Academy of Sciences, San Francisco (CAS), and the Canadian National Collection, Ottawa, Ontario, Canada (CNC).Verbatim label data are given for type specimens of all newly described taxa, with label breaks indicated by a slash (“\”), and photographs of labels from types of previously described species are also included.

Measurements. All specimens were measured electronically using a Leica M420 microscope equipped with a Syncroscopy AutoMontage Photomicroscopy system (SYNCROSCOPY, Synoptics Ltd.). Measurements for various body parts are encoded as follows: LH = length of head, measured along midline from anterior margin of labrum to the virtual line, connecting posterior supraorbital setae; WH = width of head, at level of anterior supraorbital setae; WPm = maximal width across pronotum; WPa = width across anterior angles of pronotum; WPp = width across posterior angles of pronotum; LP = length of pronotum from base to apex along midline; WE = width of elytra, at level of 4th umbilicate setae; LE = length of the elytra, from apex of scutellum to apex of left elytron; SBL = standardized body length, a sum of LH, LP and LE. SBL measurements are given in mm; others are presented as nine ratios: mean widths-WH/WPm and WPm/WE and body parts-WPa/WPp, WPm/WPp, WPm/LP, WE/LE, LE/SBL, WE/SBL and LP/LE. All values are given as mean ± standard deviation.

Illustrations. Digital photographs of the dorsal habitus of new species were taken with the AutoMontage system using a Leica M420 microscope. Line drawings of selected body parts were made using a camera lucida on an Olympus BX 50 microscope. Scanning electron micrographs were made with coating on an ESEM FEI Quanta 200.

Dissections. Most of the specimens we examined were already dissected by previous investigators, and genitalia of these specimens were preserved in microvials with glycerol, pinned beneath the specimen. Where necessary, dissections were made by present authors using standard technique. Genitalia were dissected from the abdomens of specimens previously softened in boiling water for 20-30 minutes. Contents of the abdomen were cleared using boiling 10% KOH for 2-3 minutes to remove internal tissues, and then washed in hot water before examination. After examination, genitalia were mounted on plastic transparent boards in dimethylhydantoin formaldehyde resin (DMHF) and pinned beneath the specimen.

Type material. The authors investigated type material of all three previously described Texan species of anillines.

Terms. Terms used in this presentation follow Sokolov et al. (2004), Sokolov and Carlton (2008) and Sokolov (2013).

Species ranking. Species recognition is in accordance with our previous approach (Sokolov et al. 2004).

Descriptions. The scheme of descriptions follows that of Ball and Shpeley (2005, 2009).

## Taxonomic treatment

### Anillinus

Taxon classificationAnimaliaColeopteraCarabidae

Casey, 1918

Anillinus Casey, 1918: 167. For generic synonymy, see Bousquet (2012: 699).

#### Type species.

*Anillinus carolinae* Casey, 1918.

#### Recognition.

All examined specimens of Texan Anillina are characterized by the following combination of characters: Head totally covered with microsculpture (Fig. 2A–C) comprised of irregular, nearly isodiametric sculpticells. Anterior margin of labrum and clypeus straight. Frontal area flat with minute tubercle (ft) medially near frontoclypeal suture. Fronto-lateral carinae distinct and long. Primary head setae include a pair of clypeal (cs), a pair of frontal (fs) and two pairs of supraorbital (ass and pss) setae. Labium (Fig. 3A–B) with mental tooth; mentum and submentum separated by mental-submental suture (ms). Glossal sclerite (gsc) with distinct paraglossae (pg) laterally and with two setae apically. Maxillary palps (Fig. 2A–C) with short 4th palpomere (mp4), which is 0.2–0.3 length of palpomere 3 (mp3). Pronotum (Fig. 2D–F) of various proportions, totally covered with microsculpture (which is pronounced in most specimens, but present only as very fine microlines visible only at a certain angle in some specimens), with two long primary lateral setae (middle, ls, and basal, bs) on each side. Elytra (Fig. 2G–J) totally covered with microsculpture, with basal margination (bm) distinct and long, with scutellar, three discal, apical, and the series umbilicata setae. Scutellar and discal setae of similar size and approximately three times longer than surrounding vestiture. In most species, last two (8th and 9th) pores (eo8 and eo9) of umbilicate series much closer to each other than 7th (eo7) pore is to 8^th^ (not so only in *Anillinus depressus* (Jeannel)). Abdomen with ventrite 5 of male with two and of female with four setae along the posterior margin. Aedeagi of males of all examined species with two parameres (Fig. 6), the typical configuration for most Anillina.

The arrangement of discal setae of elytra and the presence of two parameres in male aedeagi allow us to place all investigated species into the genus *Anillinus*. Beetles at hand vary in habitus from slightly to markedly elongate (WE/SBL ≤ 0.38), possess pronota with a rather narrow basal margin (WPa/WPp ≥ 1.00), and are completely covered with microsculpture dorsally (Fig. 2A–J). This combination of features allows us to place all of them in group 1 of endogean species of *Anillinus* (Sokolov et al. 2004).

#### A key for identification of adults of the genus *Anillinus* from Texas

**Table:** 

1	Larger beetles on average (ABL range 1.60–2.00 mm); pronotum with basal margin more or less straight, posterior angles not shifted forward; elytral umbilicate series of pores with 8^th^ and 9^th^ pores geminate (Fig. 2G–I,eo8 and eo9)	2
–	Smaller beetles on average (ABL < 1.50 mm); pronotum with basal margin oblique laterally, posterior angles shifted forward (Fig. 1C); elytral umbilicate series of pores with 8^th^ and 9^th^ pores disassociated, 8^th^ pore situated approximately equidistant from 7^th^ and 9^th^ pores (Fig. 17, p. 57, Jeannel 1963a)	*Anillinus depressus* (Jeannel)
2	Body markedly elongate, subparallel, with head, pronotum and elytra of approximately equal width (Fig. 4E–F); width ratios of body parts: Hd/Prnt>0.78, Prt/Eltr>0.85; pronotum more elongate (Wpm/LP<1.25), with shallow basilateral sinuation before the nearly rectangular (90–100°) posterior angles (Fig. 2E–F)	3
–	Body slightly elongate, more ovoid, with narrower head and more oval and wider elytra (Fig. 5A–D); width ratios of body parts: Hd/Prnt<0.78, Prt/Eltr<0.85; pronotum more transverse (Wpm/LP>1.27), lateral margins varied	4
3	Apex of elytron widely concave with a long curved spine on the outer margin of incision (Fig. 2J, 5E); female spermatheca with distal part of cornu (dpc) only slightly dilated (Fig. 7G)	*Anillinus acutipennis* Sokolov & Reddell, sp. n.
–	Apex of elytron EITHER truncate OR shallowly incised, without spine laterally (Fig. 2H–I, 5F); female spermatheca with distal part of cornu (dpc) markedly dilated (Fig. 7E)	*Anillinus forthoodensis* Sokolov & Reddell, sp. n.
4	Pronotum with microsculpture distinct at any angle, with lateral margins more or less rectilinearly constricted towards slightly obtuse (100–110°) posterior angles (as in Fig. 2D); beetle from the territories to the north of Bexar County	5
–	Pronotum with fine microsculpture visible on disc only at certain angles, lateral margins with shallow basilateral sinuation before the nearly rectangular (90–100°) posterior angles (Fig. 1B, Fig. 5B); beetle from Bexar County	*Anillinus sinuatus* (Jeannel)
5	Male with abdominal ventrites modified as in Fig. 3D; male metafemora with small spine at middle of posterior edge (Fig. 4D); median lobe of male aedeagus without protuberance on dorsal margin (Fig. 6A); spermatheca of female with distal part of cornu (dpc) markedly dilated (Fig. 7C); habitus as in Fig. 1A	*Anillinus affabilis* (Brues)
–	Male with abdominal ventrites simple (Fig. 3C); spermatheca of female varied	6
6	Metafemora of male modified, triangularly dilated medially (Fig. 4E); median lobe of male aedeagus with protuberance (dp) on dorsal side, without spinose ventral sclerite (Fig. 6G, J); spermatheca of female with distal part of cornu markedly dilated (Fig. 7D); habitus as in Fig. 5C	*Anillinus wisemanensis* Sokolov & Kavanaugh, sp. n.
–	Metafemora of male unmodified, fusiform (Fig. 4G); median lobe of male with dorsal side evenly rounded, without dorsal protuberance extended beyond the general contour, and with large spinose ventral sclerite (vs) in the inner sac (Fig. 6K); spermatheca of female with distal part of cornu only slightly dilated (Fig. 7F); habitus as in Fig. 5D	*Anillinus comalensis* Sokolov & Kavanaugh, sp. n.

### Anillinus acutipennis

Taxon classificationAnimaliaColeopteraCarabidae

Sokolov & Reddell sp. n.

http://zoobank.org/7E00D63B-F269-4A2A-97F9-45D5ABFDAB07

Figs 2C, F, J , 3B , 5E , 7G , 8

#### Type material.

HOLOTYPE, a female, deposited in CAS, point-mounted, dissected, labeled: \ TX: Bell Co., Talking Crows Cave, Fort Hood, 4.V.2006, J. Fant, M. Reyes \ Texas Memorial Museum Invertebrate Zool Coll #45.781 \ Holotype *Anillinus acutipennis* Sokolov & Reddell 2014 [red label] \. CAS Type No. 18870 \. PARATYPES: 2 females, both dissected; one, in CNC, labeled: \ TX: Bell Co., Hidden Pit Cave, Fort Hood, 27.X.2007, J. Reddell, M. Reyes \ Texas Memorial Museum Invertebrate Zool Coll #60.107 \; one, in TMM, labeled: \ TX: Hays Co., Wiseman Sink, 10mi, 2.IV.1995, A. G. Grubbs, C. Jordan \ Texas Memorial Museum Invertebrate Zool Coll #27.148 \. Both paratypes also labeled: \ Paratype *Anillinus acutipennis* Sokolov & Reddell 2014 [yellow label] \.

#### Type locality.

U.S.A., Texas, Bell County, Fort Hood area.

#### Etymology.

The specific epithet is a Latinized adjective in the masculine form and is derived from the Latin adjective *acutus* meaning "acute, sharpened" and the Latin noun *penna* meaning “feather, wing”. The epithet refers to the spinose apex of elytron in members of the new species.

#### Recognition.

Adults of this new species are distinguished easily from those of other Texan species of the genus by the following combination of external characters: markedly elongated habitus, distinctly elongate pronotum with shallow basolateral sinuation, and incised elytral apex with a distinct spine.

#### Description.

Medium-sized for genus (SBL range 1.62–1.84 mm, mean 1.74±0.116 mm, n=3).

Habitus. Body form (Fig. 5E) subdepressed, subparallel, markedly elongate (WE/SBL 0.33±0.009), head large for genus compared to pronotum (WH/WPm 0.80±0.006), pronotum wide in comparison to elytra (WPm/WE 0.88±0.013).

Color. Body rufotestaceous, appendages testaceous.

Microsculpture. Distinct over all dorsal surfaces of head, pronotum and elytra, with slightly transverse polygonal meshes of more or less scaly appearance on elytra.

Head. Labium (Fig. 3B). Mental tooth present; mentum and submentum separated by suture. Glossal sclerite with distinct paraglossae laterally and with two setae apically.

Prothorax. Pronotum (Fig. 2F) relatively long (LP/LE 0.42±0.016) and markedly elongated (WPm/LP 1.23±0.080), with lateral margins shallowly sinuate and moderately constricted posteriorly (WPm/WPp 1.28±0.020). Anterior angles indistinct, posterior angles nearly rectangular (90–100°). Width between anterior and posterior angles of approximately equal length (WPa/WPp 1.02±0.024). Basal margin straight.

Elytra (Fig. 2J). Widely depressed along suture, comparatively short (LE/SBL 0.57±0.004) and narrow for genus (WE/LE 0.59±0.020), with traces of 4-5 striae. Humeri distinct, rounded, in outline forming obtuse angle with longitudinal axis of body. Lateral margins subparallel, slightly divergent at basal fourth, evenly rounded to apex in apical fourth, without subapical sinuation. Vestiture of elytra short (less than one-third length of discal setae). Apex of elytron deeply emarginate, the notch with a distinct spine laterally.

Male unknown.

Female genitalia. Spermatheca with distal part of cornu only slightly dilated, gradually tapered to proximal part. Nodulus short, ramus undifferentiated (Fig. 7G).

#### Geographical distribution.

This species has been found only in two remote areas of Bell (Lampasas Cut Plain) and Hays (Balcones Fault Zone) Counties, Texas (Fig. 8, white stars).

#### Way of life.

This species has been found only in caves. The specimens from caves in Bell County were taken in darkness on the underside of rocks shallowly embedded in soil.

#### Relationships.

In general habitus and in the modified apex of the elytra, members of this new species closely resemble *Anillinus forthoodensis* adults, described below; however, they are more similar to members of *Anillinus comalensis*, also described below, in the shape of the spermatheca. In the absence of males, the relationships of this species to the other Texan anillines is unclear. The presence of a spine on the lateral margin of the apex of the elytron is unique to this species among the known *Anillinus* species of Texas.

### Anillinus affabilis

Taxon classificationAnimaliaColeopteraCarabidae

(Brues)

**Figure 1.**
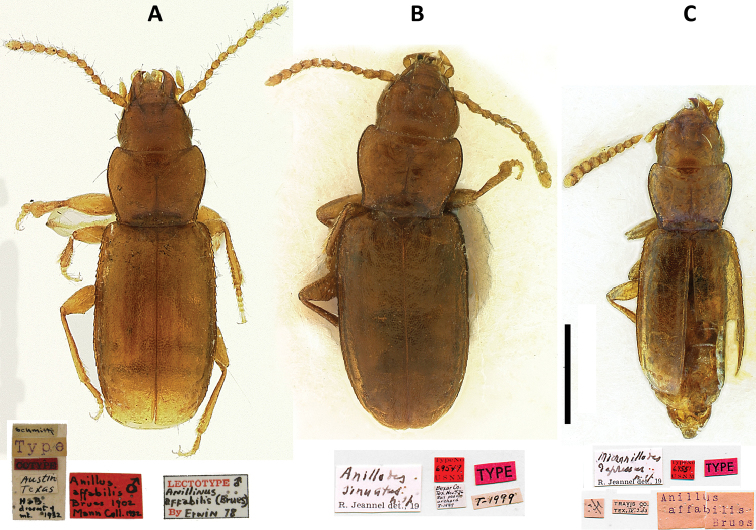
Images of type specimens of previously described Texas Anillina species. **A** *Anillus affabilis* Brues, lectotype; **B** *Anillodes sinuatus* Jeannel, holotype **C** *Micranillodes depressus* Jeannel, holotype. Scale = 0.5 mm.

Figs 1A , 3D , 4D , 5A , 6A–C , 7B–C , 8

Anillus affabilis Brues, 1902: 366. Leng 1920
Anillinus affabilis (Brues), Jeannel 1937; Erwin and House 1978; Bousquet 2012
Anillodes affabilis (Brues), Jeannel 1963a

#### Type material.

*Anillus affabilis* Brues, 1902: 366. Lectotype (Fig. 1A), designated by Erwin and House (1978: 233), a male, in NMNH, glued on a hair, dissected, and labeled: \ schmitti [handwritten] \ Type [typed] \ COTYPE [typed red label] \ Austin Texas [handwritten] \ HSB dissect of int. 1932 [handwritten] [these first five labels mounted on a single card] \ Anillus affabilis Brues 1902 Mann Coll. 1932 [red handwritten label with male symbol] \ LECTOTYPE [male symbol] Anillinus affabilis (Brues) By Erwin 78 \. Two paralectotypes: one female, also in NMNH, labeled: \ Austin Texas [printed] \ E. caecum [handwritten] \ type [typed with blue ink] \ TYPE [typed red label] \ ADP 115719 \ Anillodes affabilis Brues R, Jeannel det. 19 \ USNM TM #2067637 \ Paralectotype *Anillus affabilis* Brues recognized by Sokolov & Kavanaugh 2014 [printed yellow label] \; one male, in MCZ, labeled: \ Austin Tex \ Type [white label] \ H. C. Fall Collection \ Anillus affabilis Brues \ Paralectotype *Anillus affabilis* Brues recognized by Sokolov & Kavanaugh 2014 [printed yellow label]

#### Notes on nomenclature and types.

In his original description, Brues (1902) noted that he had examined three specimens of this species, all from Austin, Texas. In 1978, the paralectotype specimens noted above were unknown to Erwin and House (1978: 233) but they have been located in the MCZ and NMNH since then.

#### Recognition.

Females of *Anillinus affabilis* are practically indistinguishable from females of *Anillinus wisemanensis*, described below. From adults of other Texan species of the genus, those of *Anillinus affabilis* can be distinguished by the following combination of external characters: large size, comparatively narrow and transverse pronotum with rectilinearly constricted lateral margins, rather wide and long elytra with rounded apices; and males can be further distinguished by the minute spine on the posterior edge of metafemora and shape of the median lobe.

#### Redescription.

Medium-sized for genus (SBL range 1.73–1.96 mm, mean 1.87±0.120 mm, n=3).

Habitus. Body form (Figs 1A, 5A) subdepressed, subparallel, slightly elongate (WE/SBL 0.38±0.010), head normally proportioned for genus (WH/WPm 0.76±0.026), pronotum moderately narrow in comparison to elytra (WPm/WE 0.78±0.007).

Color. Body rufotestaceous or brunneorufous, appendages testaceous.

Microsculpture. Distinct over all dorsal surfaces of head, pronotum and elytra, with slightly transverse polygonal meshes of more or less scaly appearance on elytra.

Prothorax. Pronotum of normal length (LP/LE 0.39±0.012) and slightly transverse for genus (WPm/LP 1.31±0.016), lateral margins almost rectilinear and moderately constricted posteriorly (WPm/WPp 1.32±0.008). Anterior angles indistinct, posterior angles slightly obtuse (100–110°). Width between anterior and posterior angles of equal length (WPa/WPp 1.00±0.026). Basal margin almost straight.

Elytra. Widely depressed along suture, of normal length (LE/SBL 0.58±0.008) and typical width for genus (WE/LE 0.66±0.029), with traces of 4-5 striae. Humeri distinct, rounded, in outline forming right angle with longitudinal axis of body. Lateral margins subparallel, slightly divergent at basal fourth, evenly rounded to apex in apical fourth, without subapical sinuation. Vestiture of elytra short (less than one-third length of discal setae). Apex of elytron rounded.

Legs. Male protarsomere 1 markedly dilated apico-laterally with rows of adhesive setae ventrally. Male hind legs modified: metafemora with minute tooth at middle along posteroventral margin (Fig. 4D).

Abdomen. Abdominal ventrites 3 and 4 of males each with a pair of protuberances at the places of setal attachments (Fig. 3D). Last visible abdominal ventrite of male slightly depressed (Fig. 3D).

Male genitalia. Median lobe of aedeagus (Fig. 6A) with short basal lobe, long arcuate shaft, and strongly enlarged apex, broadly rounded at tip. Dorsal margin strongly sclerotized along almost all its length. Ventral margin enlarged along entire length from apex to basal orifice, bearing numerous poriferous canals. Dorsal sclerite in form of a semicircular blade-like structure with characteristic basal prolongations. Without distinct ventral sclerites, but with few sclerotized fields in apicoventral area. Dorsal membraneous field with small spines located dorsally from dorsal sclerite. Enlarged apical area of median lobe with a dark spine-like structure. Right paramere slightly enlarged, long and narrow, with numerous (>8) long setae (Fig. 6C), approximately equal in length to length of the paramere. Left paramere slightly enlarged apically and greatly so basally, with translucent keel and large lateral process comparable in size to paramere itself (Fig. 6B), without long setae.

Female genitalia. Gonocoxite 2 unguiform (Fig. 7B), rather long, with moderately curved blade and narrowly rounded apex, with nematiform and two ensiform setae, the lateral of which is thicker than the medial one. Laterotergite with 9-10 setae. Spermatheca (Fig. 7C, sp) with distal part of cornu (dpc) markedly dilated and abruptly narrowed to the proximal part (ppc). Nodulus (n) short, ramus undifferentiated.

#### Geographical distribution.

This species is known only from Travis County, Texas (Fig. 8, black triangle), in the vicinity of the Balcones Fault Zone. In addition to the lectotype and paralectotype, we have examined a total of five specimens (3 males and 2 females), all dissected: one male and one female labeled: \ TX: Travis Co., Tooth Cave, 24.V.1992, J. Reddell \ Texas Memorial Museum Invertebrate Zool Coll #27.145 \; one male and one female, in CNC, labeled: \ TX: Travis Co., Tooth Cave, 6.VI.1992, J. Reddell \ Texas Memorial Museum Invertebrate Zool Coll #27.146 \; one male, in TMM, labeled: \ TX: Travis Co., Three-Holer Cave, 18.VIII.1990, J. Reddell, M. Reyes \ Texas Memorial Museum Invertebrate Zool Coll #27.144 \.

#### Way of life.

All newly collected beetles were found in caves on the underside of rocks shallowly embedded in soil. Specimens of the type series were sifted from ant nests (Brues 1902).

#### Relationships.

The shape of the spermatheca in females and the armature of the internal sac and type of ventral enlargement of the median lobe in males suggest a likely relationship with *Anillinus forthoodensis*.

### Anillinus comalensis

Taxon classificationAnimaliaColeopteraCarabidae

Sokolov & Kavanaugh sp. n.

http://zoobank.org/39FE83DE-4E8E-4A42-9008-2C6E3EF8EFC9

Figs 4G , 5D , 6K–M , 7F , 8

#### Type material.

HOLOTYPE, a male, deposited in CAS, point-mounted, dissected, labeled: \ TX: Comal Co., 7mi W New Braunfels, 27.I.1995, A. G. Grubbs \ Texas Memorial Museum Invertebrate Zool Coll #27.151 \ Holotype *Anillinus comalensis* Sokolov & Kavanaugh 2014 [red label] \ CAS Type No. 18871 \. PARATYPES: 2 females, one in CNC and one in TMM, both dissected, labeled same as holotype, except each with the following label: \ Paratype *Anillinus comalensis* Sokolov & Kavanaugh 2014 [yellow label].

#### Type locality.

U.S.A., Texas, Comal County, 7mi W New Braunfels.

#### Etymology.

The specific epithet is a Latinized adjective in the masculine form based on the name of Comal County, from which the new species is described.

#### Recognition.

Adults of this new species are distinguished from those of other Texan species of the genus by the following combination of external characters: slightly ovoid and narrow elytra, rather short and transverse pronotum and rounded apex of elytron; and males are further distinguished by the unmodified hind legs.

#### Description.

Medium-sized for genus (SBL range 1.72–1.86 mm, mean 1.78±0.072 mm, n=3).

Habitus. Body form (Fig. 5D) subdepressed, slightly ovate, slightly elongate (WE/SBL 0.37±0.002), head normally proportioned for genus (WH/WPm 0.76±0.015), pronotum rather narrow in comparison to elytra (WPm/WE 0.80±0.015).

Color. Body rufotestaceous, appendages testaceous.

Microsculpture. Distinct over all dorsal surfaces of head, pronotum and elytra, with slightly transverse polygonal meshes of more or less scaly appearance on elytra.

**Figure 2.**
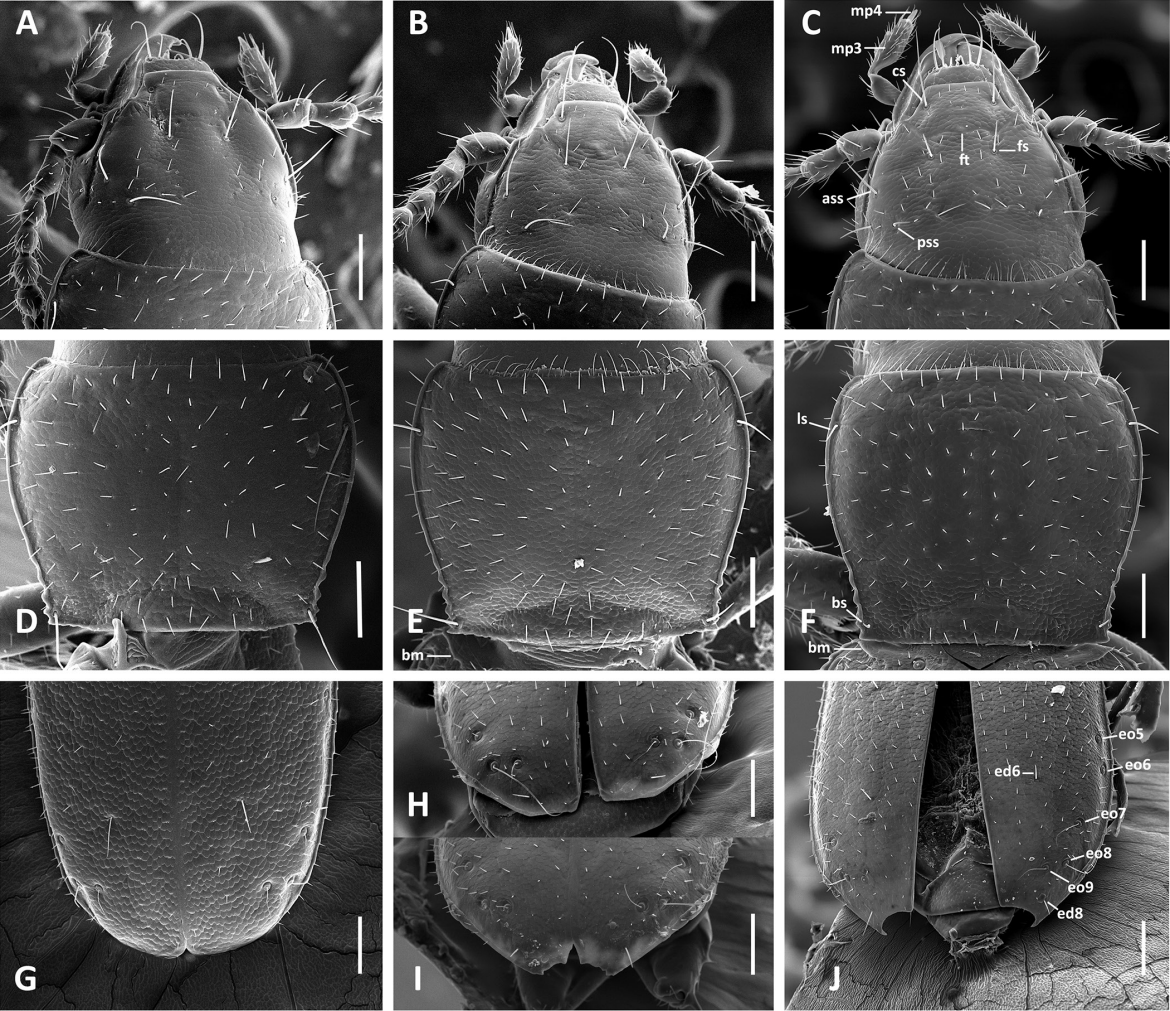
SEM images of body parts, dorsal aspect, of *Anillinus* species. **A–C** Head: **A** *Anillinus wisemanensis* (TEXAS, Hays County, Wiseman Sink) **B** *Anillinus forthoodensis* (TEXAS, Bell County, Talking Crows Cave) **C** *Anillinus acutipennis* (TEXAS, Bell County, Talking Crows Cave) **D–F** Pronotum: **D** *Anillinus wisemanensis* (TEXAS, Hays County, Wiseman Sink) **E** *Anillinus forthoodensis* (TEXAS, Bell County, Talking Crows Cave) **F** *Anillinus acutipennis* (TEXAS, Bell County, Talking Crows Cave) **G–J** Apical half of elytra: **G** *Anillinus wisemanensis* (TEXAS, Hays County, Wiseman Sink) **H** *Anillinus forthoodensis* (TEXAS, Bell County, Bell Cave) **I** *Anillinus forthoodensis* (TEXAS, Bell County, Talking Crows Cave) **J** *Anillinus acutipennis* (TEXAS, Bell County, Talking Crows Cave). ass – anterior supraorbital seta; bm – basal margination; bs – basilateral pronotal seta; cs – clypeal seta; ed6 – 3d discal seta; ed8 – apical seta; eo5-9 setae from the umbilical series; fs – frontal seta; pss – posterior supraorbital seta; ft – frontal tubercle; ls – midlaterall pronotal seta; mp3 – maxillary palpomere 3; mp4 – maxillar palpomere 4. Scale bars = 0.1 mm.

Prothorax. Pronotum (as in Fig. 2D) relatively short (LP/LE 0.37±0.022) and markedly transverse (WPm/LP 1.31±0.046), with lateral margins almost rectilinear and moderately constricted posteriorly (WPm/WPp 1.29±0.027). Anterior angles indistinct, posterior angles slightly obtuse (100–110°). Width between anterior and posterior angles of equal length (WPa/WPp 1.00±0.037). Basal margin almost straight.

Elytra (as in Fig. 2G). Widely depressed along suture, comparatively long (LE/SBL 0.61±0.010) and narrow for genus (WE/LE 0.61±0.010), with traces of 4-5 striae. Humeri distinct, rounded, in outline forming right angle with longitudinal axis of body. Lateral margins subparallel, slightly divergent at basal fourth, evenly rounded to apex in apical third, without subapical sinuation. Vestiture of elytra short (less than one-third length of discal setae). Apex of elytron rounded.

Legs. Male protarsomere 1 markedly dilated apico-laterally with adhesive setae ventrally. Male hind legs unmodified (Fig. 4G).

Abdomen. Ventrite 5 of male without depression.

Male genitalia. Median lobe of aedeagus (Fig. 6K) with short basal lobe (bl), long arcuate shaft (sh), and enlarged apex, rounded at tip. Dorsal margin strongly sclerotized along basal two thirds of the shaft length. Ventral margin enlarged in apical half, with numerous poriferous canals on wall of shaft medially. Dorsal sclerite (ds) in form of a curved blade-like structure with very short basal prolongations. Ventral sclerite (vs) in form of semicurcular plate narrow in middle and abruptly widened at both ends, surfaces of which are totally spinose. Distinct spines are absent from internal sac. Enlarged apical area (aa) of median lobe with a dark spine-like structure (ss). Right paramere enlarged, very short and wide with numerous (>8) long setae (Fig. 6M) with length approximately equal to length of paramere. Left paramere of normal shape (Fig. 6L), without long setae.

Female genitalia. Spermatheca with distal part of cornu only slightly dilated, gradually tapered to the proximal part. Nodulus short, ramus undifferentiated (Fig. 7F).

#### Geographical distribution.

This species is known only from the type locality in the New Braunfels area, Comal County, Texas (Fig. 8, white diamond), in the Balcones Fault Zone.

#### Way of life.

The type specimens were taken from the underside of limestone rocks in talus.

#### Relationships.

The unusual structure of the male median lobe and unmodified hind legs of males suggest that this species is not closely related to any of the other Texan anillines.

### Anillinus depressus

Taxon classificationAnimaliaColeopteraCarabidae

(Jeannel)

Figs 1C , 8

Micranillodes depressus Jeannel 1963a: 58. Holotype, a female, in NMNH, glued on cardboard and labeled as in Fig. 1C.
Anillinus depressus (Jeannel), Bousquet 2012

#### Recognition.

Adults of *Anillinus depressus* are distinguished easily from those of other Texan species of the genus by the following combination of external characters: small size, markedly elongated habitus, laterally oblique pronotal basal margin and simple (i.e., neither truncated nor incised elytral apex).

#### Redescription.

Small-sized for genus (SBL 1.45 mm).

Habitus. Body form (Fig. 1C) subdepressed, subparallel, elongate (WE/SBL 0.34), head large for genus compared to pronotum (WH/WPm 0.83), pronotum wide in comparison to elytra (WPm/WE 0.85).

Color. Body rufotestaceous, appendages testaceous.

Microsculpture. Present over all dorsal surfaces of head, pronotum and elytra, but much finer on head and pronotum than on elytra.

Head. Labium with mental tooth present; mentum and submentum separated by suture.

Prothorax. Pronotum relatively long (LP/LE 0.41) and slightly transverse (WPm/LP 1.29), with lateral margins almost rectilinear and strongly constricted posteriorly (WPm/WPp 1.33). Anterior angles indistinct, posterior angles slightly obtuse (100–110°). Width between posterior angles less than between anterior angles (WPa/WPp 1.10). Basal margin oblique laterally, so posterior angles noticeably shifted forward.

Elytra. Widely depressed along suture, of normal length (LE/SBL 0.57) and narrow for genus (WE/LE 0.61), with traces of 4-5 striae. Humeri distinct, rounded, in outline forming right angle with longitudinal axis of body. Lateral margins subparallel, slightly divergent at basal fourth, evenly rounded to apex in apical third, without subapical sinuation. Umbilicate series with 9 pores, 8^th^ and 9^th^ of which not geminate. Apex of elytron rounded with distinct sutural angle.

Genitalia not examined.

#### Geographical distribution.

The single known specimen of this species was collected in Travis County, Texas (Fig. 8, black diamond), in the vicinity of the Balcones Fault Zone.

#### Way of life.

No precise data provided in original description or on labels associated with the holotype.

#### Relationships.

Contrary to Jeannel’s description (1963a), the type specimen possesses a distinct mental-submental suture on the labium and normal, not shortened, elytra [although the apical half of the right elytron is missing]. Chaetotaxy of the elytra cannot be investigated because of their poor condition, but Jeannel cited and illustrated three discal setae for the specimen. If so, then this specimen can be treated as a representative of *Anillinus*, as was done by Bousquet (2012). Configuration of the last pores of the umbilicate series may be of low importance for a separate generic status of the specimen, because it is known that this character is variable within “good” genera of anillines (Giachino and Vailati 2011). The small size and reduction of microsculpture on the foreparts of the body of its members make this species morphologically distinct from other Texan anillines. Perhaps, this combination of characters reflects adaptations for living in a rather different environment in comparison with the other species.

### Anillinus forthoodensis

Taxon classificationAnimaliaColeopteraCarabidae

Sokolov & Reddell sp. n.

http://zoobank.org/A5899D9D-93A5-45A0-BC9E-E13A8A7EBED6

**Figure 3.**
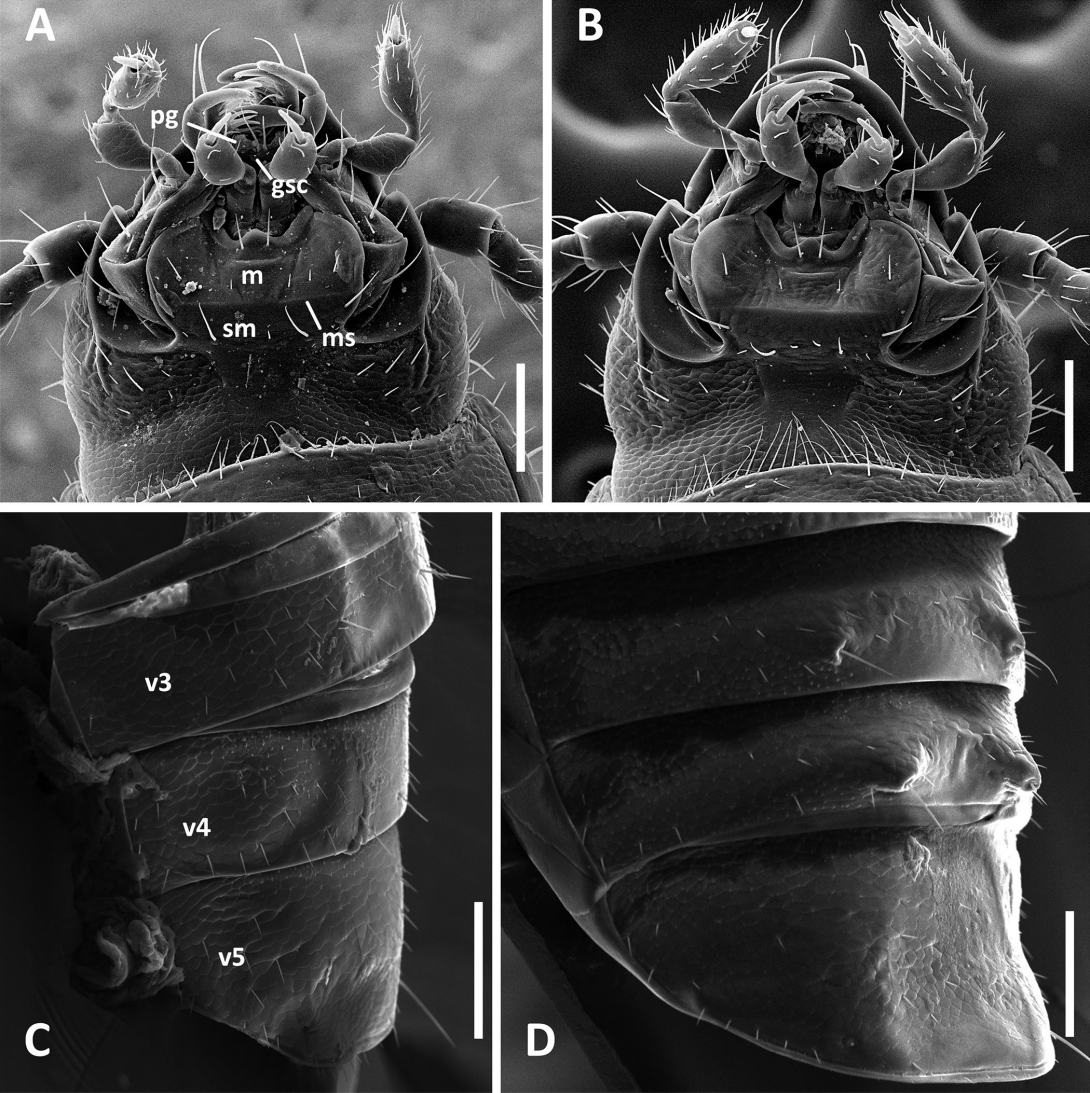
SEM images of structural features of *Anillinus* species. **A–B** Head capsule, ventral aspect: **A** *Anillinus forthoodensis* (TEXAS, Bell County, Talking Crows Cave) **B** *Anillinus acutipennis* (TEXAS, Bell County, Talking Crows Cave) **C–D** Abdominal ventrites 3-5, males, latero-ventral aspect: **C** *Anillinus forthoodensis* (TEXAS, Bell County, Talking Crows Cave) **D** *Anillinus affabilis* (TEXAS, Travis County, Tooth Cave). gsc – glossal sclerite; m – mentum; ms – mental-submental suture; pg – paraglossa; sm – submentum; v3-v5 – abdominal ventrites. Scale bars = 0.1 mm.

**Figure 4.**
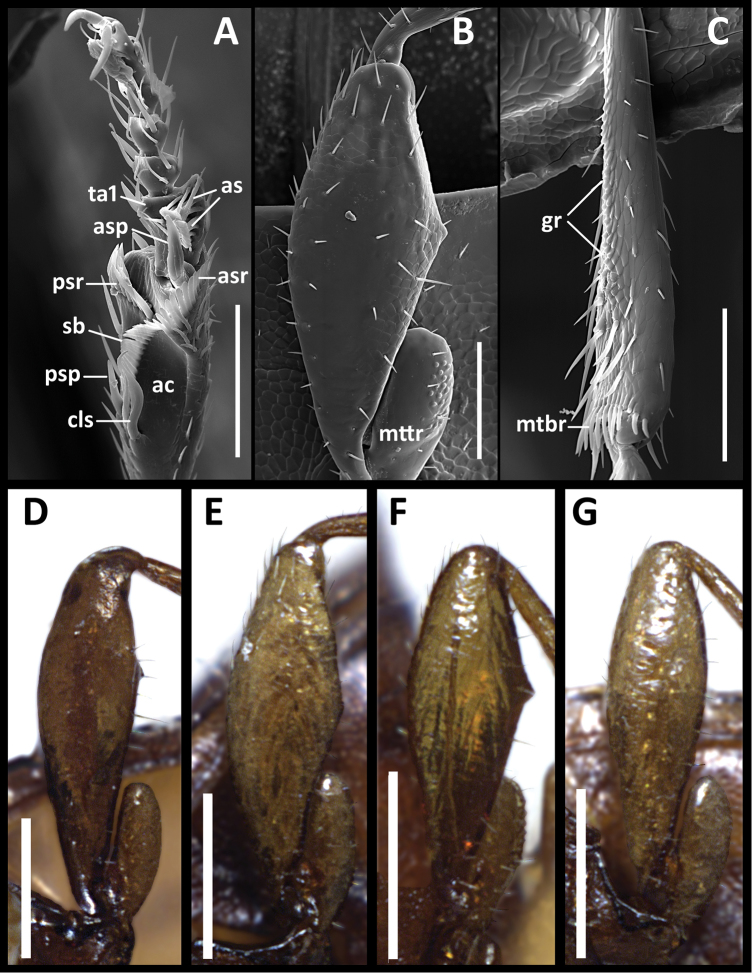
Structural features of legs of males of *Anillinus* species. *Anillinus forthoodensis* (TEXAS, Bell County, Talking Crows Cave): **A** left protibia **B** left metafemur **C** left metatibia **D–G** Left metafemora: **D** *Anillinus affabilis* (TEXAS, Travis County, Tooth Cave) **E** *Anillinus wisemanensis* (TEXAS, Hays County, Wiseman Sink) **F** *Anillinus forthoodensis* (TEXAS, Bell County, Talking Crows Cave) **G** *Anillinus comalensis* (TEXAS, Comal County, 7mi W New Braunfels). ac – antennal cleaner; as – articulo-setae; asp – anterior spur; asr – anterior setal row; cls – clip seta; gr – granulation; mtbr – metatibial brush; mttr – metatrochanter; psp – posterior spur; psr – posterior setal row; sb – setal band; ta1 – tarsomere 1. Scale bars: **A–C** = 0.1 mm;. **D–G** = 0.2 mm.

**Figure 5.**
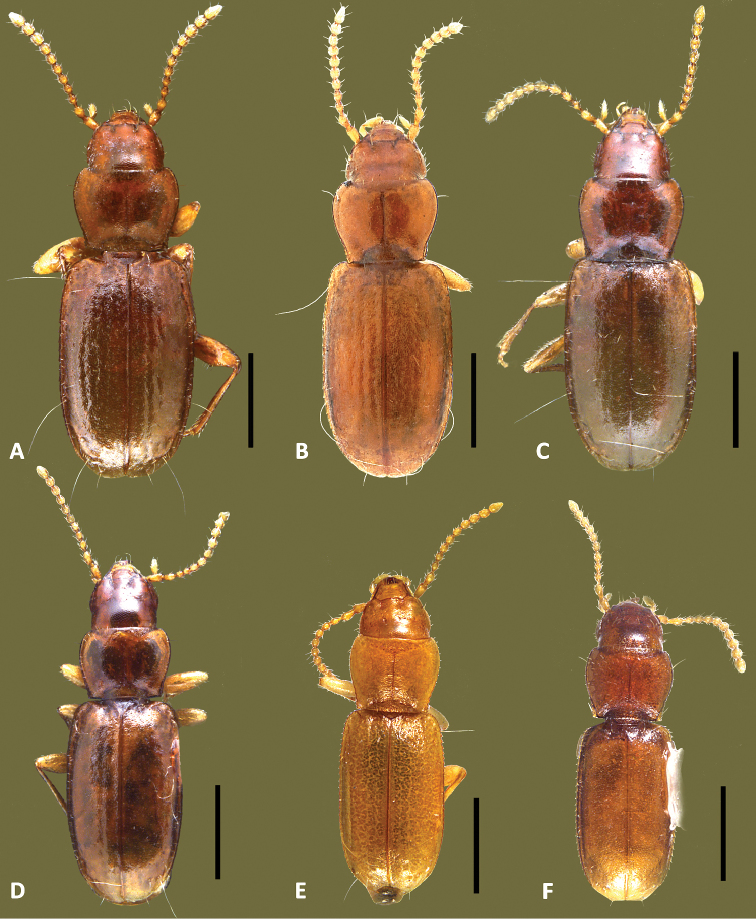
Habitus images of *Anillinus* species. **A** *Anillinus affabilis* (TEXAS, Travis County, Tooth Cave) **B** *Anillinus sinuatus* (TEXAS, Bexar County) **C** *Anillinus wiseman* ensis (TEXAS, Hays County, Wiseman Sink), holotype; holotype **D** *Anillinus comalensis* (TEXAS, Comal County, 7mi W New Braunfels), paratype **E** *Anillinus acutipennis* (TEXAS, Bell County, Hidden Pit Cave), paratype **F** *Anillinus forthoodensis* (TEXAS, Bell County, Talking Crows Cave), holotype. Scale bar = 0.5 mm.

**Figure 6.**
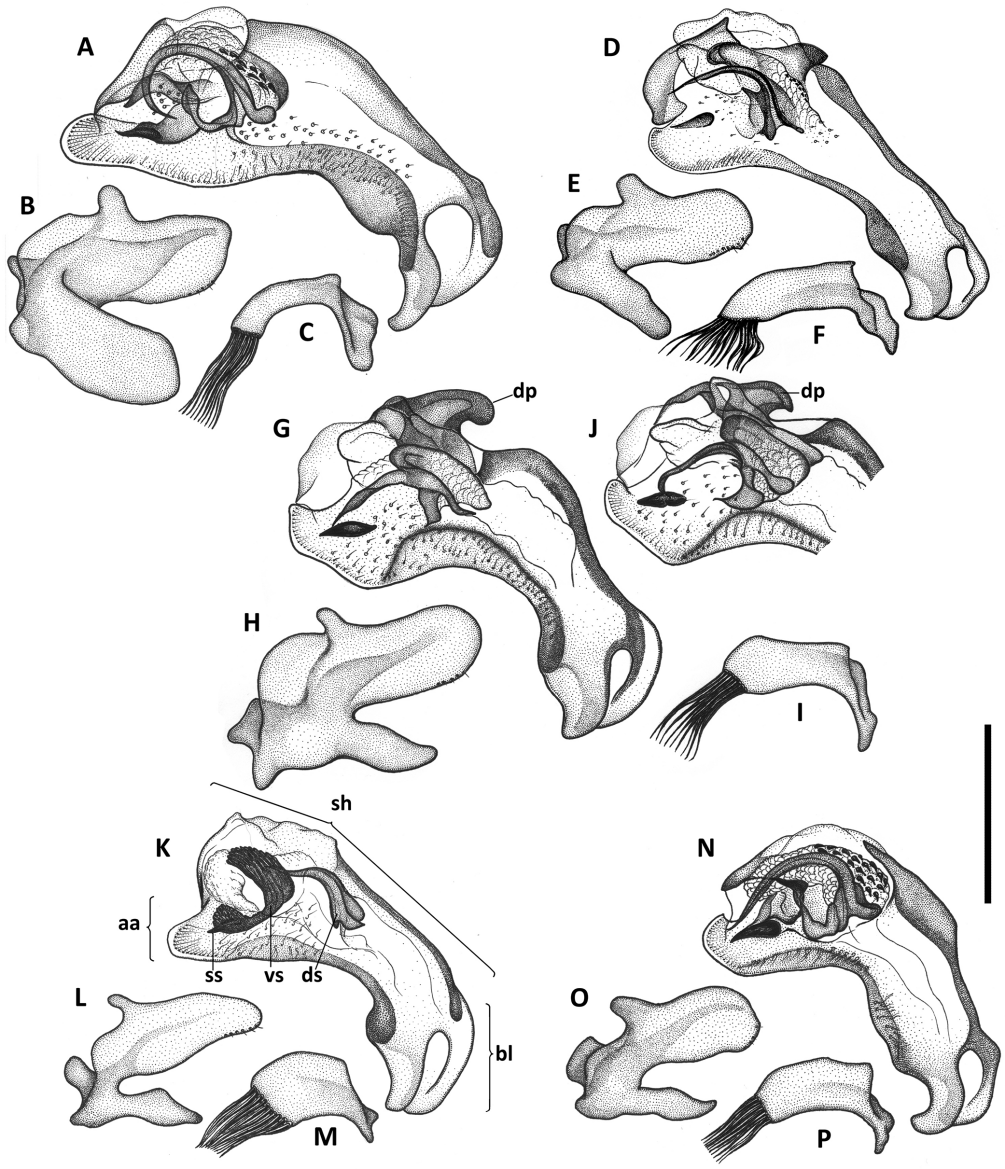
Illustrations of male aedeagus of *Anillinus* species. *Anillinus affabilis* (TEXAS, Travis County, Tooth Cave): **A** median lobe, right lateral aspect **B** left paramere, left lateral aspect **C** right paramere, right lateral aspect. *Anillinus sinuatus* (TEXAS, Bexar County) **D** median lobe, right lateral aspect **E** left paramere, left lateral aspect **F** right paramere, right lateral aspect. *Anillinus wisemanensis* (TEXAS, Hays County, Wiseman Sink) **G** median lobe, right lateral aspect **H** left paramere, left lateral aspect **I** right paramere, right lateral aspect. *Anillinus wisemanensis* (TEXAS, Bell County, Talking Crows Cave) **J** median lobe, right lateral aspect. *Anillinus comalensis* (TEXAS, Comal County, 7mi W New Braunfels) **K** median lobe, right lateral aspect **L** left paramere, left lateral aspect **M** right paramere, right lateral aspect. *Anillinus forthoodensis* (TEXAS, Bell County, Talking Crows Cave) **N** median lobe, right lateral aspect **O** left paramere, left lateral aspect **P** right paramere, right lateral aspect. aa – apical area; bl – basal lobe; dp – dorsal protuberance; ds – dorsal sclerite; sh – shaft; ss – spine-like structure; vs – ventral sclerite. Scale = 0.2 mm.

**Figure 7.**
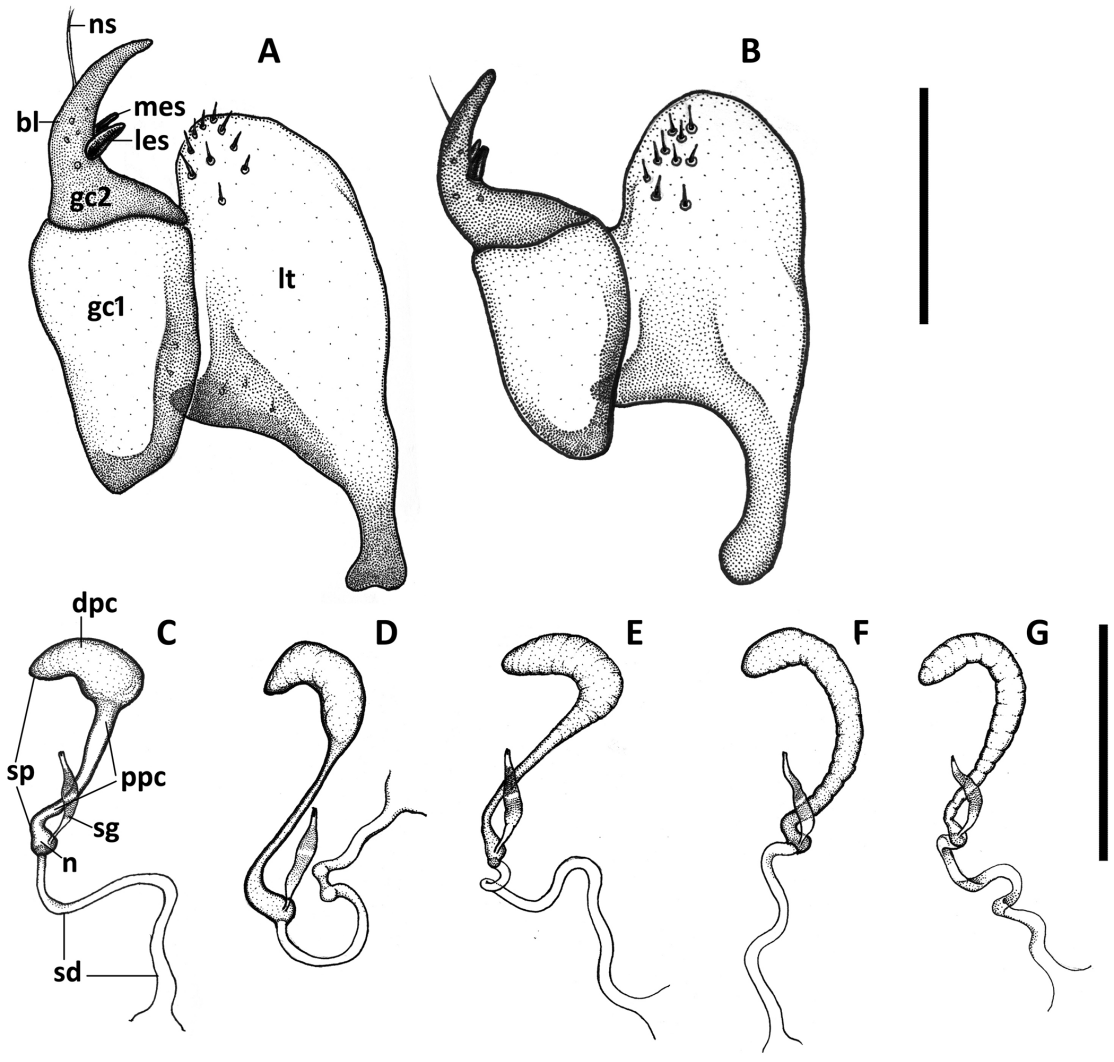
Illustrations of female genitalia of *Anillinus* species. Ovipositor sclerites: **A** *Anillinus forthoodensis* (TEXAS, Bell County, Talking Crows Cave **B** *Anillinus affabilis* (TEXAS, Travis County, Tooth Cave). Spermatheca: **C** *Anillinus affabilis* (TEXAS, Travis County, Tooth Cave) **D** *Anillinus wisemanensis* (TEXAS, Hays County, Wiseman Sink) **E** *Anillinus forthoodensis* (TEXAS, Bell County, Talking Crows Cave) **F** *Anillinus comalensis* (TEXAS, Comal County, 7mi W New Braunfels) **G** *Anillinus acutipennis* (TEXAS, Hays County, Wiseman Sink). bl – blade of gonocoxite 2, dpc – distal part of cornu; gc1 – gonoxocite 1; gc2 – gonocoxite 2; les – lateral ensiform seta; lt – laterotergite; mes – medial ensiform seta; n – nodulus; ns – nematiform seta; ppc – proximal part of cornu; sd – spermathecal duct; sg – spermathecal gland; sp – spermatheca. Scale bars: A, B = 0.1 mm; C-G = 0.2 mm.

**Figure 8.**
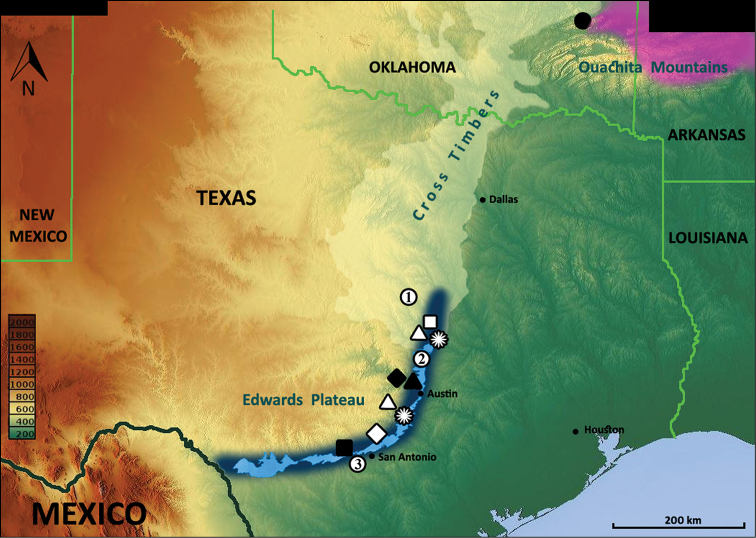
Locality records for *Anillinus* species: *Anillinus acutipennis* – white stars; *Anillinus affabilis* – black triangle; *Anillinus comalensis* – white diamond; *Anillinus depressus* – black diamond; *Anillinus forthoodensis* – white quadrangle; *Anillinus lescheni* – black circle *Anillinus sinuatus*– black quadrangle; *Anillinus wisemanensis*– white triangles; *Anillinus* spp. – white circles (1 – Bell and Coryell Counties; 2 – Williamson County; 3 – Bexar County). Violet color – range of the western *Anillinus* species in Arkansas and Oklahoma (Sokolov et al. 2004). Light blue color – recharge zone of the Edwards Aquifer, dark blue color – the Balcones Escarpment, topographic expression of the Balcones Fault Zone (Woodruff and Abbot 1986). Contour of the Cross Timbers ecological region (highlighted) were taken from Wikipedia: http://en.wikipedia.org/wiki/Cross_Timbers. Elevation scale bar is given in meters.

Figs 2B, E, H–I , 3A, C , 4A–C, F , 5F , 6N–P , 7A, E , 8

#### Type material.

HOLOTYPE, a male, deposited in CAS, point-mounted, dissected, labeled: \ TX: Bell Co., Talking Crows Cave, Fort Hood, 4.V.2006, J. Fant, M. Reyes \ Texas Memorial Museum Invertebrate Zool Coll #45.781 \ Holotype *Anillinus forthoodensis* Sokolov & Reddell 2014 [red label] \ CAS Type No. 18872. PARATYPES: 4 males and 3 females; two males and one female, in TMM, labeled same as holotype; one male, in CNC, labeled: \ TX: Bell Co., Talking Crows Cave, Fort Hood, 2 June 2005, J. Fant, J. Reddell, M. Reyes \ Texas Memorial Museum Invertebrate Zool Coll #38.153 \; one female, in TMM, labeled: \ TX: Bell Co., Nolan Creek Cave, Fort Hood, 27.IV.2007, J. Fant, J. Reddell \ Texas Memorial Museum Invertebrate Zool Coll #55.333 \; one male and one female, in TMM, labeled: \ TX: Bell Co., Bell Cave, Fort Hood, 4 March 2010, J. Fant \ Texas Memorial Museum Invertebrate Zool Coll #70.872 \. All paratypes also labeled: \ Paratype *Anillinus forthoodensis* Sokolov & Reddell 2014 [yellow label] \.

#### Type locality.

U.S.A., Texas, Bell County, Fort Hood area.

#### Etymology.

The specific epithet is a Latinized adjective in the masculine form based on Fort Hood, the U. S. military post located in Texas, from the surroundings of which the new species is described.

#### Recognition.

Adults of this new species are distinguished easily from those of other Texan species of the genus by the following combination of external characters: small size, markedly elongate habitus, distinctly elongate pronotum with shallowly sinuate lateral margins, and truncate apex of elytron.

#### Description.

Medium-sized for genus (SBL range 1.65–1.73 mm, mean 1.69±0.040 mm, n=4).

Habitus. Body form (Fig. 5F) subdepressed, subparallel, markedly elongate (WE/SBL 0.33±0.010), head large for genus compared to pronotum (WH/WPm 0.79±0.022), pronotum wide in comparison to elytra (WPm/WE 0.88±0.031).

Color. Body rufotestaceous, appendages testaceous.

Microsculpture. Distinct over all dorsal surfaces of head, pronotum and elytra, with slightly transverse polygonal mesh of more or less scaly appearance on elytra.

Head. Labium (Fig. 3A) with mental tooth; mentum and submentum separated by suture. Glossal sclerite with distinct paraglossae laterally and with two setae apically.

Prothorax. Pronotum (Fig. 2E) relatively long (LP/LE 0.43±0.027) and markedly elongate (WPm/LP 1.21±0.024), with lateral margins shallowly sinuate and moderately constricted posteriorly (WPm/WPp 1.29±0.016). Anterior angles indistinct, posterior angles nearly rectangular (90–100°). Width between anterior and posterior angles of approximately equal length (WPa/WPp 1.03±0.012). Basal margin slightly convex.

Elytra (Fig. 2H–I). Widely depressed along suture, of normal length (LE/SBL 0.56±0.010) and narrow for genus (WE/LE 0.59±0.022), with traces of 4-5 striae. Humeri distinct, rounded, in outline forming obtuse angle with longitudinal axis of body. Lateral margins subparallel, slightly divergent at basal fifth, evenly rounded to apex in apical fourth, without subapical sinuation. Vestiture of elytra short (less than one-third length of discal setae). Apex of elytron truncate (Fig. 2H, 6 specimens out of 7 investigated) or shallowly emarginate (Fig. 2I, 1 female from 7 investigated).

Legs. Male protarsomere 1 markedly dilated apico-laterally with two rows of adhesive setae ventrally (Fig. 4A). Male hind legs modified: trochanters with many minute bumps scattered across ventral surface (Fig. 4B), metafemora triangularly dilated along posteroventral margin with a small tooth at tip of dilation (Fig. 4B, F), and metatibiae with granulate posterior margin (Fig. 4C).

Abdomen. Ventrite 5 of male with medial depression (Fig. 3C).

Male genitalia. Median lobe of aedeagus (Fig. 6N) with short basal lobe, long rectangularly bent shaft, and enlarged apex, broadly rounded at tip. Dorsal margin strongly sclerotized along almost all its length. Ventral margin enlarged along entire length to basal orifice, with numerous poriferous canals. Dorsal sclerite in form of a semicircular blade-like structure with characteristic basal prolongations. Without distinct ventral sclerites. Dorsal membranous field with numerous small spines located dorsally from dorsal sclerite. Enlarged apical area of median lobe with a dark spine-like structure. Right paramere enlarged, long and wide with numerous (>8) long setae (Fig. 6P), with length approximately two-thirds of length of the paramere. Left paramere wide, slightly enlarged apically and basally (Fig. 6O), without long setae.

Female genitalia. Gonocoxite 2 (Fig. 7A) unguiform (gc2), rather long, with slightly curved blade (bl) and narrowly rounded apex, with nematiform (ns) and two ensiform setae, with the lateral (les) of these thicker than the medial (mes). Laterotergite (lt) with 9–10 setae. Spermatheca with distal part of cornu markedly dilated. Nodulus short, ramus undifferentiated (Fig. 7E).

#### Geographical distribution.

This species is known only from several caves distributed in the Fort Hood area, Bell County, Texas (Fig. 8, white quadrangle), Lampasas Cut Plain.

#### Way of life.

This species has been found only in caves. Specimens were taken in darkness on the underside of rocks shallowly embedded in soil.

#### Relationships.

The medially depressed abdominal ventrite 5, the enlargement of the ventral margin of the median lobe and the presence of small spines on the dorsal membranous field in the internal sac of both *Anillinus forthoodensis* and *Anillinus affabilis* males suggest that they are the closest relatives among the Texan anillines.

### Anillinus sinuatus

Taxon classificationAnimaliaColeopteraCarabidae

(Jeannel)

Figs 1B , 5B , 6D–F , 8

Anillodes sinuatus Jeannel 1963a: 57. Holotype, a female, deposited in NMNH, glued on cardboard and labeled as in Fig. 1B.
Anillinus sinuatus (Jeannel), Bousquet 2012

#### Recognition.

Adults of *Anillinus sinuatus* are distinguished from those of other Texan species of the genus by the following combination of external characters: large size, pronotum with long and shallow basilateral sinuations and microsculpture on pronotum much finer than on head and elytra; and males are further distinguished by the minute spine on the posterior edge of metafemora and shape of the median lobe.

#### Redescription.

Medium-sized for genus (SBL range 1.81–1.84 mm, mean 1.83±0.019 mm, n=2).

Habitus. Body form (Fig. 1B, 5B) subdepressed, subparallel, slightly elongate (WE/SBL 0.37±0.007), head normally proportioned for genus (WH/WPm 0.77±0.009), pronotum rather narrow in comparison to elytra (WPm/WE 0.81±0.008).

Color. Body rufotestaceous, appendages testaceous.

Microsculpture. Distinct over all dorsal surfaces of head and elytra. Pronotum with much finer microsculpture, almost indistinct on disc and only at certain angles.

Prothorax. Pronotum of normal length (LP/LE 0.40±0.004) and slightly transverse for genus (WPm/LP 1.31±0.019), lateral margins with long and shallow sinuation before posterior angles, moderately constricted posteriorly (WPm/WPp 1.33±0.024). Anterior angles indistinct, posterior angles nearly rectangular (90–100°). Width between anterior and posterior angles of approximately equal length (WPa/WPp 1.03±0.020). Basal margin almost straight.

Elytra. Widely depressed along suture, of normal length (LE/SBL 0.58±0.008) and typical width for genus (WE/LE 0.64±0.003), with traces of 5-6 striae. Humeri distinct, rounded, in outline forming right angle with longitudinal axis of body. Lateral margins subparallel, slightly divergent at basal fourth, evenly rounded to apex in apical fourth, without subapical sinuation. Vestiture of elytra short (less than one-third length of discal setae). Apex of elytron rounded.

Legs. Male protarsomere 1 markedly dilated apico-lateraly with rows of adhesive setae ventrally. Male hind legs modified: metafemora with minute tooth at middle along posteroventral margin.

Abdomen. Abdominal ventrites of males unmodified.

Male genitalia. Median lobe of aedeagus (Fig. 6D) with short basal lobe, long curved shaft, and enlarged apex, broadly rounded at tip. Dorsal margin strongly sclerotized along almost all its length, with rather large protuberance directed backwards and situated before apical orifice. Ventral margin enlarged only apically, where it bears numerous poriferous canals. Dorsal sclerite in form of a semicircular blade-like structure with short basal prolongations. Without distinct ventral sclerites. Dorsal membraneous field with two very small spines located dorsally from dorsal sclerite. Enlarged apical area of median lobe with a dark spine-like structure. Right paramere slightly enlarged, long, of moderate width with numerous (>8) long setae (Fig. 6F), their length shorter than length of paramere. Left paramere enlarged apically and basally, where it forms a translucent convex keel (Fig. 6E), without long setae.

Female genitalia. Not investigated.

#### Geographical distribution.

This species is known only from Bexar County, Texas (Fig. 8, black quadrangle), in the vicinity of the Balcones Fault Zone. In addition to the holotype, we have examined a total of 2 specimens (one male and the fragmentary remains of one female): one male (dissected), in NMNH, labeled: \ BexarCo. Tex. May 5 1938 T-11135 38-8191 \ From soil of peach orch. \ T-11135 \ USNM \ *Anillinus sinuatus* n. sp R. Jeannel det., 19 \ *Anillodes sinuatus* J. det. T.L.Erwin 96 \; one female (presumably the same species, represented only by two legs [right middle and hind legs]) on points, in NMNH, labeled: \ Bexar Co. Tex. Feb. 9. 1938 T-9056 38-2676 \ From soil of peach orchard \ T-9056 \ USNM \.

#### Way of life.

All specimens at hand were extracted from soil during sampling surveys in peach orchards.

#### Relationships.

The armature of the internal sac and the presence of the dorsal protuberance on the median lobe suggest a close relationship with *Anillinus wisemanensis*, described below.

### Anillinus wisemanensis

Taxon classificationAnimaliaColeopteraCarabidae

Sokolov & Kavanaugh sp. n.

http://zoobank.org/A2AB931C-A7FA-4F55-BCB7-473FABB599E4

Figs 2A, D, G , 4E , 5C , 6G–I , 7D , 8

#### Type material.

HOLOTYPE, a male, deposited in CAS, point-mounted, dissected, labeled: \ TX: Hays Co., Wiseman Sink, 28.IV.1995, A. G. Grubbs \ Texas Memorial Museum Invertebrate Zool Coll #27.149 \ Holotype *Anillinus wisemanensis* Sokolov & Kavanaugh 2014 [red label] \ CAS Type No. 18873 \. PARATYPES: 1 male and 3 females, all dissected; one male and one female, in TMM, labeled: \ TX: Hays Co., Wiseman Sink No 2, 10mi W San Marcos, 22.IV.1995, A. G. Grubbs \ Texas Memorial Museum Invertebrate Zool Coll #27.150 \; one female, in TMM, labeled: \ TX: Hays Co., Wiseman Sink, 10mi, 2.IV.1995, A. G. Grubbs, C. Jordan \ Texas Memorial Museum Invertebrate Zool Coll #27.148 \; one female, in CNC, labeled: \ TX: Hays Co., Wiseman Sink, 30.IV.1995, A. G. Grubbs \ Texas Memorial Museum Invertebrate Zool Coll #27.147 \. All paratypes also labeled: \ Paratype *Anillinus wisemanensis* Sokolov & Kavanaugh 2014 [yellow label] \.

#### Type locality.

U.S.A., Texas, Hays County, Wiseman Sink.

#### Etymology.

The specific epithet is a Latinized adjective in the masculine form based on Wiseman Sink, the caves from which the type specimens were obtained.

#### Recognition.

Females of *Anillinus wisemanensis* are virtually indistinguishable from those of *Anillinus affabilis*. Adults of this new species are distinguished from those of other Texan species of the genus by the following combination of external characters: only slightly transverse pronotum with rectilinearly constricted lateral margins, comparatively wide and short elytra, and rounded elytral apices; and males are further distinguished by the triangularly dilated metafemora and distinctive dorsal protrusion of the median lobe.

#### Description.

Medium-sized for genus (SBL range 1.68–1.90 mm, mean 1.77±0.110 mm, n=4), specimens from Bell County slightly larger (SBL range 1.81–1.93 mm, n=2).

Habitus. Body form (Fig. 5C) subdepressed, subparallel, slightly elongate (WE/SBL 0.36±0.010), head normally proportioned for genus (WH/WPm 0.76±0.015), pronotum moderately wide in comparison to elytra (WPm/WE 0.82±0.012).

Color. Body brunneorufous, appendages testaceous.

Microsculpture. Distinct over all dorsal surfaces of head, pronotum and elytra, with slightly transverse polygonal meshes of more or less scaly appearance on elytra.

Prothorax. Pronotum (Fig. 2D) of normal length (LP/LE 0.39±0.017) and of normal proportions for genus (WPm/LP 1.28±0.032), lateral margins almost rectilinear and moderately constricted posteriorly (WPm/WPp 1.29±0.025). Anterior angles indistinct, posterior angles slightly obtuse (100–110°). Width between anterior and posterior angles of equal length (WPa/WPp 1.01±0.028). Basal margin almost straight.

Elytra (Fig. 2G). Widely depressed along suture, of normal length (LE/SBL 0.58±0.007) and rather narrow for genus (WE/LE 0.61±0.013), but specimens from Bell County with slightly wider elytra (WE/LE 0.64±0.004), traces of 4-5 striae evident. Humeri distinct, rounded, in outline forming right angle with longitudinal axis of body. Lateral margins subparallel, slightly divergent at basal fourth, evenly rounded to apex in apical third, without subapical sinuation. Vestiture of elytra short (less than one-third length of discal setae). Apex of elytron rounded.

Legs. Male protarsomere 1 markedly dilated apico-lateraly with rows of adhesive setae ventrally. Male hind legs modified: metafemora triangularly dilated along posteroventral margin (Fig. 4E), and metatibiae with granulated posterior margin.

Abdomen. Ventrite 5 of male unmodified.

Male genitalia. Median lobe of aedeagus (Fig. 6G) with short basal lobe, almost rectangularly bent long shaft, and strongly enlarged apex, characteristically angulate ventrally and narrowly rounded at tip. Dorsal margin strongly sclerotized along almost all its length, with large protuberance directed backwards and situated before apical orifice. Ventral margin enlarged along all length, but most widely in apical half and then gradually tapered to basal orifice. Numerous poriferous canals on the ventral margin and medial walls of the shaft. Dorsal sclerite in form of a curved blade-like structure with characteristic basal prolongations. Without distinct ventral sclerites or spines. Enlarged apical area of median lobe with a dark spine-like structure. Specimens from Bell County demonstrate slightly different shape of dorsal protuberance and basal prolongations of dorsal sclerite (Fig. 6J). Right paramere enlarged, long and wide with numerous (>8) long setae (Fig. 6I) approximately equal in length to length of paramere. Left paramere wide, markedly enlarged in basal area, where it forms a translucent wide keel (Fig. 6H), without long setae.

Female genitalia. Spermatheca with distal part of cornu markedly dilated. Nodulus short, ramus undifferentiated (Fig. 7D).

#### Geographical distribution.

This species is known from two widely separated localities in Bell (Lampasas Cut Plain) and Hays (Balcones Fault Zone) Counties, Texas (Fig. 8, white triangles). In addition to the type series, we have examined two male specimens, both dissected, labeled: \ TX: Bell Co., Talking Crows Cave, Fort Hood, 19.VI.2003, J. Reddell, M. Reyes \ Texas Memorial Museum Invertebrate Zool Coll #55.559 \ [These specimens correspond to the new species in all respects, except in their being of slightly larger size, in having slightly wider elytra and in the degree of development of some features of the median lobe structure].

#### Way of life.

This species has been found only in caves. Specimens from Fort Hood, Bell County, were taken in darkness from the underside of rocks shallowly embedded in soil.

#### Relationships.

The shape of the spermatheca in females and the enlarged left paramere and shape of the dorsal sclerite of the median lobe in males suggest a possible but remote relationship with *Anillinus affabilis*.

### Unidentified material

We are unable to associate definitively the following six specimens, all in TMM, with any of the seven species included above: one female labeled: \ TX: Coryell Co., Lucky Day Cave, Fort Hood, 27.VI.2009, J. Fant, J. Reddell, M. Reyes \ Texas Memorial Museum Invertebrate Zool Coll #70.013 \; one female labeled: \ TX: Bell Co., Sponge Bob Pot, Fort Hood, 17.II.2009, J. Fant, M. Warton \ Texas Memorial Museum Invertebrate Zool Coll #69.724 \; one male labeled (aedeagus lost): \ TX: Williamson Co., Lobo’s Lair, 13.IX.1991, J. Reddell & M. Reyes \ Texas Memorial Museum Invertebrate Zool Coll #27.142 \; one female labeled: \ TX: Williamson Co., Lobo’s Lair, 1.IX.1991, W. Elliot, J. Reddell, M. Reyes, M. Warton \ Texas Memorial Museum Invertebrate Zool Coll #27.126 \; one female (pronotum broken, ovipositor sclerites and spermatheca lost) labeled: \ TX: Bexar Co., Up the Creek Cave, 14.XI.1995, J. Cokendolpher, J. Reddell, M. Reyes \ Texas Memorial Museum Invertebrate Zool Coll #27.141 \; one female (in poor condition, only head, pronotum and abdominal ventrites are present, ovipositor sclerites and spermatheca lost) labeled: \Zara-3873: TX: Bexar Co., Holy Smoke Cave, 10.XII.2008, P. Sprouse, S. Zappitello \.

Females from Coryell and Bell Counties (Fig. 8, white circle with a “1”) and Bexar County (Fig. 8, white circle with a “3”) are distinguished from those of all known species in having markedly transverse pronota (WPm/LP 1.33–1.37). At the same time, specimens from Coryell and Bexar Counties possess elytra of normal width for the genus (WE/LE 0.63–0.65), whereas one female from Bexar County has narrower than typical elytra (WE/LE 0.59). The two specimens from Williamson County (Fig. 8, white circle with a “2”) belong to a new species, but in the absence of genitalia from the male specimen and the presence of only one female, we consider a description of this species premature. Additional material is needed to clarify the taxonomic position of these unidentified specimens.

### Evolutionary aspects

New findings have increased the total number of *Anillinus* species recorded from Texas to seven. The discovery of these four new species allows us to suggest some hypotheses about different aspects of their taxonomy and evolution.

The external structural features of the species lead us to include them in one morphological group of totally microsculptured endogean species, but the forms of male and female genitalia exhibit a wide range of variation, suggesting that as many as four lineages may be involved: 1, the *Anillinus affabilis-forthoodensis-sinuatus-wisemanensis* lineage; and three monobasic lineages – 2, the *Anillinus depressus* lineage, 3, the *Anillinus comalensis* lineage, and 4, the *Anillinus acutipennis* lineage. Based on the shape of the median lobe and armature of the internal sac, *Anillinus affabilis*, *Anillinus forthoodensis*, and *Anillinus wisemanensis* belong to a lineage of endogean species that is widely distributed across the geographical range of the genus. A configuration of male genitalia similar to the Texan species has been seen for the Appalachian *moseleyae*-group of species (Figs 11–12, p. 4, Sokolov 2011), *Anillinus aleyae* Sokolov & Watrous (Fig. 2, p. 540, Sokolov and Watrous 2008) from southern Missouri, and *Anillinus lescheni* Sokolov & Carlton (Fig. 30, p. 194, Sokolov et al. 2004) from eastern Oklahoma. Without a doubt, among the listed species, members of the last-named species are most similar to the Texan *Anillinus*. *Anillinus lescheni* members share with Texas specimens not only structural features of the median lobe, but also the type of metafemoral and metatibial modifications in males. Female genitalia remain mostly uninvestigated among endogean *Anillinus*, but dilation of the distal part of the cornu in the spermatheca, which is characteristic of *Anillinus affabilis*, *Anillinus forthoodensis*, and *Anillinus wisemanensis*, is unusual for litter species but also has been seen in *Anillinus aleyae* females (Fig. 5, p. 540, Sokolov and Watrous 2008) from southern Missouri. Based on similarities in the structure of hind legs of males, the shape of the median lobe and armature of the internal sac in males and the configuration of the spermatheca in females, we hypothesize that the Texan species are most closely related to and perhaps derived from species of the western Ouachita and Ozark Mountains fauna. However, this hypothesis can only be tested by a comprehensive phylogenetic analysis of genus *Anillinus*, which has not yet been undertaken.

The known distribution of Texan Anillina extends along the Balcones Fault Zone from San Antonio in the south to Georgetown in the north. Additional populations occur on the Fort Hood Military Installation in the Lampasas Cut Plains to the north (Fig. 8). With the likely exception of *Anillinus sinuatus* at San Antonio and the possible exception of *Anillinus depressus* in Travis County, all are known from caves or talus slopes in the Edwards Limestone. *Anillinus sinuatus* was collected from "peach orchards" which are not usually planted on limestone; therefore, this species may have been collected from south or east of the Balcones Fault Zone.

This virtually linear distribution of the Texan species of *Anillinus* reflects the fact that beneath the Balcones Fault Zone lie a number of underground water resources known as the Edwards-Trinity aquifer system of Texas (Ryder 1996). For endogean organisms, living more in the vertically- than horizontally-oriented world, this underground world of vast water resources provides a good opportunity to escape from the unacceptably dry climatic conditions at the above-ground periphery of their ranges. The Edwards-Trinity aquifer system harbors a diverse stygobiontic arthropod fauna (Holsinger 1967; Barr and Spangler 1992) with, for example, unique species of diving beetles (Dytiscidae) (Young and Longley 1976; Spangler and Barr 1995; Miller et al. 2009; Jean et al. 2012). Possibly, subterranean aquifer systems surrounded by highly porous and fractured limestones also harbor a special subterranean anilline fauna, which is analogous to epigean “streamside” bembidiine complexes of species and is confined to the damp rock interspaces and other crevices surrounding underground water bodies. This fauna is exceptionally difficult to sample and usually escapes sampling by investigators. However, from time to time its representatives can be collected in caves or even on the surface under rocks or in the soil after heavy rains or by using special methods of sampling.

Although the Fort Hood Military Installation is separated from the cavernous deposits of the Balcones Fault Zone by about 60 km of non-cavernous deposits, the same genera containing troglobites occur in both areas. This indicates that the ancestral species of troglobites ranged throughout the entire area. Both areas contain the same basic vegetation type and presumably were suitable habitat for the same litter-dwelling and endogean species that gave rise to the troglobites. The Fort Hood region belongs to the Lampasas Cut Plain, a subdivision of the Cross-Timbers ecological region (Gould 1975), which marks the western habitat limit of many mammals (Goetze and Nelson 2009), fishes (Hubbs 1957) and insects (Carlton 1990) and is also a transitional zone for many plants (Hatch et al. 1990) and animals whose ranges extend northward and eastward from the oak woodlands of Central Texas to the oak forests of the western Ouachita. Practically speaking, the Fort Hood region connects the modern range of Texas Anillina with the range of *Anillinus lescheni* in Oklahoma (Fig. 8), and we hypothesize that, in a previous time, probably during or just prior to the late Tertiary or early Quaternary, when the regional environment began to dry (Graham 1999), this region served as a main route for the expansion of the eastern anillines deep into the southwest. The comfortable conditions of the Balcones Fault Zone, with its rich underground water resources, appear to have triggered a new round of speciation of endogean anillines in Texas. Without doubt, unseen and unknown underground connections and barriers in the region have shaped the modern distributions of species and driven their evolution. If so, we can expect further discoveries of these hard-to-collect, enigmatic beetles both along the Balcones Fault Zone and around the Edwards-Trinity Aquifer area, and perhaps also in the Cross-Timbers region, where Anillina species have not yet been reported.

In summary, we hypothesize that four lineages of endogean, totally microsculptured *Anillinus* extended their geographical ranges from their source area in the Ouachita-Ozark Mountains to the Balconian region in central Texas. There, they encountered the cavernous Edwards-Trinity aquifer system that provided an excellent refugium as the regional climate in the late Tertiary and early Quaternary became increasingly drier, rendering the near-surface conditions too dry to sustain the life of small litter-inhabiting arthropods. Isolated within the cool damp layers of limestone penetrated by the Edwards-Trinity aquifer system, these anilline lineages differentiated, but to what extent is not known. One of them is represented by four species; each of the others might have remained monobasic, although it seems likely that they too differentiated within their Texan refugium.

## Supplementary Material

XML Treatment for Anillinus

XML Treatment for Anillinus acutipennis

XML Treatment for Anillinus affabilis

XML Treatment for Anillinus comalensis

XML Treatment for Anillinus depressus

XML Treatment for Anillinus forthoodensis

XML Treatment for Anillinus sinuatus

XML Treatment for Anillinus wisemanensis
